# Molecular Sex Identification in the Hardy Rubber Tree (*Eucommia ulmoides* Oliver) via ddRAD Markers

**DOI:** 10.1155/2020/2420976

**Published:** 2020-05-16

**Authors:** Wencai Wang, Guoqian Yang, Xin Deng, Fengqing Shao, Yongquan Li, Wei Guo, Hong Liang, Xianzhi Zhang

**Affiliations:** ^1^Science and Technology Innovation Center, Guangzhou University of Chinese Medicine, Guangzhou 510405, China; ^2^Institute of Clinical Pharmacology, Guangzhou University of Chinese Medicine, Guangzhou 510405, China; ^3^School of Agriculture and Biology, Shanghai Jiao Tong University, China; ^4^College of Horticulture and Landscape Architecture, Zhongkai University of Agriculture and Engineering, Guangzhou 510225, China

## Abstract

*Eucommia ulmoides*, also known as the industrially and medicinally important hardy rubber tree, is the sole species of Eucommiaceae. Nevertheless, its dioecious property hinders sex recognition by traditional morphological observation at very early developmental stages, thus inhibiting breeding and economic cropping. In this study, double-digest restriction site-associated DNA sequencing (ddRAD-seq) was applied to screen sex-linked molecular markers for sex identification and investigation of the sex determination system in 20 male and female *E. ulmoides* individual plants, respectively. In consequence, five candidate male-specific loci but no female-specific loci were predicated among the 183,752 male and 147,122 female catalogue loci by bioinformatics analysis. Subsequent PCR (polymerase chain reaction) amplification and Sanger sequencing examinations were performed on another 24 individuals, 12 for each sex, from a separate population. One ideal sex-linked locus, MSL4, was identified among the five putative male-specific loci that were found using ddRAD data. MSL4 is 479 bp in length and highly conserved in all the male individuals, suggesting its feature of being stable and repeatable. Our results also indicated that the sex of *E*. *ulmoides* is likely determined genetically. In short, this study provides a consistent and reproducible ddRAD marker (MSL4) that is able to discriminate male from female seedlings in *E*. *ulmoides*, which will be valuable for rapid breeding practice and better commercial production of this economically important tree.

## 1. Introduction

*Eucommia ulmoides* Oliver, belonging to the monotypic family Eucommiaceae [1], is an economically important dioecious tree endemic to southern and central China [2]. It is a well-known Tertiary relict tree species and widely cultivated as a medicinal plant in China for more than 2,000 years [3]. In recent years, this species has been called as the hardy rubber tree due to the high productivity of gutta, i.e., TPI (trans-1,4-polyisoprene) in the whole plant [4–6]. Because of its wide adaptability in the subtropical and temperate regions, *E*. *ulmoides* is therefore to potentially supersede the commonly known para rubber tree (*Hevea brasiliensis* in Euphorbiaceae) that is restricted in the tropical zones [7, 8]. However, sex identification of the young seedlings of *E*. *ulmoides* is rather difficult for its dioecious sexual system and long life cycle (up to 8-10 years), which limits the efficiency of breeding for improving the gutta (Eu-rubber) yield or other agronomic traits [9].

Dioecy represents the sex separation in distinct individuals, which resembles the majority of mammals that include individuals of males and females [10]. Only about 6% of the flowering plants, i.e., 15,600 species in 987 genera of 175 families, however, are dioecious [11]. In general, there are three kinds of sex determination system via sex chromosomes that have been revealed in dioecious plants: XY, ZW, and XO [12]. XY sex chromosome system has heterogametic sex in males (XY) while females are homogametic XX, which is determined in *Actinidia chinensis* [13], *Asparagus officinalis* [14], *Carica papaya* [15], and *Silene latifolia* [16]. In contrast, ZW sex chromosome system features female heterogamety (ZW) and male homogamety (ZZ), such as in *Populus trichocarpa* [17]. Some other species, e.g., *Rumex acetosa*, evolve an XO sex determination system, in which the sex of one individual is determined by the X-to-autosome ratio [18]. Until now, heteromorphic sex chromosomes are only verified in 39 plants by reliable cytogenetic and/or molecular evidence [19]. The dioecious hardy rubber tree *E*. *ulmoides* has a diploid genotype with a basic composition of 2*n* = 34 chromosomes and circa 1.2 Gb of genome size [20]. Nevertheless, it remains uncertain whether *E*. *ulmoides* has a genetic sex determination or heteromorphic sex chromosome [21, 22], leading to the difficulty and unreliability to identify the sex of *E*. *ulmoides* during the vegetative growth phase via karyotype [23].

Moreover, with the advent of genomics, sex-determining genes are emerging in several plants [24]. For instance, in kiwifruit (*Actinid*ia spp.), Y-encoded genes *SyGI* and *FrBy* act independently as the suppressor of feminization (SuF) and the maintenance of male (M) [25]. In persimmon (*Diospyros* spp.), a Y-encoded pseudogene *OGI* is sufficient for the expression of androecium and repression of gynoecium through encoding a small RNA and targeting its autosomal counterpart gene *MeGI* [26]. Two Y-encoded factors, named *SOFF* and *aspTDF*, have been discovered in garden asparagus (*A*. *officinalis*) for the expression of maleness and repression of female development [27]. Nonrecombining male-specific regions including many genes have also been characterized in *C*. *papaya* [28], *P*. *trichocarpa* [29], and *S*. *latifolia* [30]. Recently, one putative MADS box APETALA3-like gene, with male-biased expression, was identified to be probably involved in the sex determination in *E*. *ulmoides* based on transcriptomic analysis [31]. Nevertheless, to our knowledge, the sex determination mechanism in *E*. *ulmoides* is still in the dark to date.

Sexual dimorphisms for instance morphological and physiological differences between the genders have been reported in dioecious plants [32, 33]. The gutta yields display a remarkable sexual dimorphism between the leaves of male and female trees of *E*. *ulmoides* [21]. The fruit accumulates the highest content of gutta among the leaves, barks, roots, fruits, and seeds of *E*. *ulmoides* [7]. An optimal proportion of 6-8% of males is considered to be sufficient for providing sperm in the *Eucommia* orchard [23]. It is thus essential to develop a simple, accurate, and effective method for sex identification of *E*. *ulmoides* long before phenomorphological features appear to satisfy the optimal sex ratio for gutta production.

Ideal sex-linked molecular markers can assist to select off the low-value male seedlings and to keep pistillate trees at the juvenile stage, consequently to enlarge economic benefits of the orchard, as that applied in the kiwifruit [34] and sea buckthorn (*Hippophae rhamnoides*) [35]. Restriction site-associated DNA sequencing (RAD-seq) and its modified version double-digest RAD-seq (ddRAD-seq), high-throughput sequencing methods developed on the basis of next-generation sequencing, enable the discovery of tens of thousands of molecular markers for nonmodel species in ecological and evolutionary studies [36, 37]. Considering that RAD-seq/ddRAD-seq has been successfully used to identify sex-linked DNA sequences in dioecious plants, e.g., in *Excoecaria agallocha* [38], kiwifruit [39], and *S*. *latifolia* [40], in this study, we employed a slightly modified ddRAD (*Mi*ddRAD) technique [37] to develop sex-specific DNA markers for gender identification of *E*. *ulmoides* during the vegetative growth phase.

## 2. Materials and Methods

### 2.1. Plant Materials

**Figure 1:**
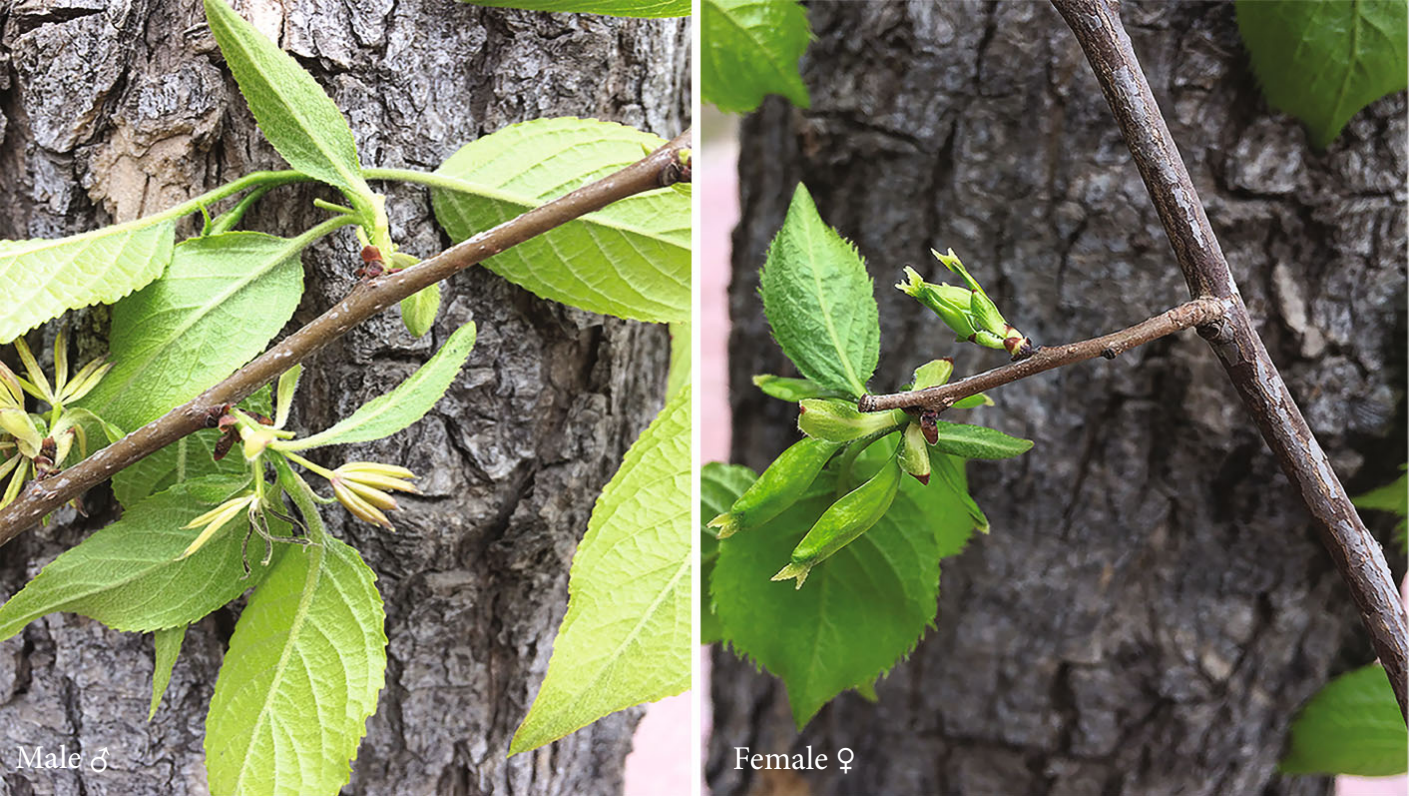
Male (♂) and female (♀) individuals of *Eucommia ulmoides* distinguished by flowers.

A total of 20 female and 20 male trees from a population of *E*. *ulmoides* cultivated on the campus of Northwest A&F University, Yangling, Shaanxi, China (34°16′56^″^N, 108°04′27^″^E) were sampled for ddRAD marker development. Their sexes were identified by flower traits during the flowering season (April in 2017), when discrimination of the staminate and pistillate genotypes was decided (Figure 1). For each individual, fresh healthy leaves were collected before being immersed into liquid nitrogen immediately and then stored at -80°C until DNA isolation. In addition, we sampled 12 male and 12 female individuals from Henan Normal University in Xinxiang, Henan, China (34°41′33^″^N, 113°40′16^″^E) in April 2018 as a separate population to validate the sex-specific markers. The distance between these two sampling points is about 650 km.

### 2.2. DNA Isolation, ddRAD Library Construction, and Illumina Sequencing

Genomic DNA was isolated from the leaf samples using the CTAB method [41]. The quality of DNA was monitored on 1.0% (m/*v*) agarose gel and NanoPhotometer® spectrophotometer (Implen, CA, USA). We constructed a ddRADseq library for the 20 male individuals and 20 female individuals following the *Mi*ddRAD protocol [37]. In brief, *AvaII*+*MspI* enzyme pair was used to digest the purified genomic DNA and ligated the barcoded adaptors. Then, the DNA samples were pooled and fragments with 500-600 bp in length were selected for PCR amplification. The enriched DNA was purified and measured by gel and Qubit 2.0 Fluorometer (Life Technologies, CA, USA). Finally, the library was sequenced on the Illumina HiSeq 2500 platform at Novogene, Beijing, China, to generate paired-end (PE) reads with 150 nucleotides in length.

### 2.3. ddRAD-seq Data Analysis

Raw reads were demultiplexed using the process_radtags algorithm in Stacks software version 1.24 [42, 43]. Average sequence quality per read was estimated by FastQC version 0.11.3 [44], and adapter reads were determined using Cutadapt 1.9.1 [45]. Reads of low quality (below a Phred score of Q10), with ambiguous barcodes (the presence of mismatch), or missing the restriction site, and the adaptor reads were removed to produce clean data. All the clean reads were truncated to a final length of 135 base pairs (bp), excluding the barcode and low-quality nucleotides at the 3′ end, for subsequent analyses.

The *ustacks* module in Stacks was used firstly to merge short-read sequences into tags/loci at the set of –*m* 10, -*M* 3, in which *m* is the minimum number of identical reads required to create a stack and *M* is the maximum distance (in nucleotides) allowed between stacks [38]. Then, a catalog was built by the *cstacks* program (−*n* 3) for all these individuals, in which *n* is the maximum number of mismatches allowed between the loci in the catalog. Finally, the output of *sstacks* (matches.tsv) from each sample was used to identify putative sex-specific loci by the R script [46]. The ddRAD locus that was present in ≥*N*-1 individuals of one sex (*N* is the total number of individuals of one gender) while absent in all individuals of the other sex was defined as putative sex-specific markers [38].

Since each member of the PE reads was treated as an independent locus in the above analysis, we further investigated if there were cases where two of the predicted sex-specific ddRAD loci represented two members of a PE read pair. In addition, clean reads of the opposite sex were mapped to each of the chosen loci by the Linux grep command to rule out the possibility of any exact matches in sequence to the selected locus. These two steps can narrow down the number of candidate markers [38].

### 2.4. PCR Validation of Putative Sex-Specific ddRAD Loci

We subsequently conducted PCR to validate these putative sex-specific ddRAD loci based on the additional 24 individuals (12 for each sex) collected from one separate population in Henan. The forward and reverse primers were designed with Primer3 [47] and anchored the sequences of the putative sex-linked ddRAD loci (Table S1). We used the volume of 25 *μ*L for PCR amplification reaction: 12.5 *μL* PCR mix (Tiangen Biotech, Beijing, China), 2.0 *μ*L random decamer primers (25 *μ*mol·*μ*L^−1^), 0.5 *μ*L genomic DNA (100 ng·*μ*L^−1^), and 10.0 *μ*L ddH_2_O. The following PCR profile was used: an initial denaturation at 94°C for 3 min followed by 30 cycles of denaturation (30 s at 94°C), annealing (30 s at 59°C) and extension (1 min at 72°C), and a final extension 72°C for 5 min. The PCR amplification products were finally examined on a 1.0% agarose gel. Each PCR experiment was repeated twice to validate both the reliability and stability.

### 2.5. Sanger Sequencing of the Verified ddRAD Marker

In addition, for the verified ddRAD marker that was only amplified via PCR in males but not in females (male-specific locus MSL4), we sequenced the band by Sanger sequencing on ABI 3730X at Sangon Biotech, Shanghai, China. We used the MSL4-F primer (Table S1) as the sequenced primer, and all the 12 male individuals used in PCR validation were sequenced. The obtained 12 sequences were aligned to produce a consensus sequence in Geneious v11.1 [48], which was searched against the *in silico* predicted sex-specific ddRAD loci by blastn with *e* value < 10^−5^ [49] to confirm their origin of same genomic position. The identified ddRAD marker was also blast to the NCBI nucleotide database (https://www.ncbi.nlm.nih.gov/) to investigate their potential functional information. Furthermore, we run the local blastn by setting the marker MSL4 as query and *Eucommia* genome scaffolds (https://bigd.big.ac.cn/gwh/Assembly/13/show) [20] as database.

## 3. Results

### 3.1. ddRAD Sequencing and Sex-Linked Loci Development

A total of 40 individuals of *E*. *ulmoides*, with each sex having 20 individuals, were sequenced according to the ddRAD-seq protocol. About 2-3 Gb data were produced for each individual, reaching a total of 180,645,228 paired-end (PE) reads (read length = 150 bp, 100 Gb). After demultiplexing and quality filtering, we got a total of 178,740,749 reads (read length = 135 bp, 54 Gb). The number of reads in the 40 samples varied from 2,186,096 to 6,742,116 (Table 1). The *ustacks* analysis recovered 20,807 to 95,993 ddRAD tags in these samples. Using *cstacks*, we obtained 183,752 loci and 147,122 loci in males and females, respectively.

Through screening the *sstacks* output (matches.tsv), we preliminarily detected 11 candidate male-linked ddRAD loci that were present in 19/20 males and none in the 20 females. Based on further checking, if these loci represented a pair of PE reads and had no exact match of female clean reads, we finally identified five loci as putative male-specific ddRAD markers, which were denoted as MSL1, MSL2, MSL3, MSL4, and MSL5 (Table 2). Given that the DNA fragments of ddRAD-seq are about 500 bp in length and should be tightly linked, we considered each marker as one independent genomic locus. Interestingly, there were no candidate female-linked ddRAD loci that were found in our analysis. The ddRAD-seq data have been deposited in the Sequence Read Archive (SRA) of the NCBI database, with the accession number PRJNA607161.

### 3.2. Male-Specific ddRAD Marker Examination

**Figure 2:**
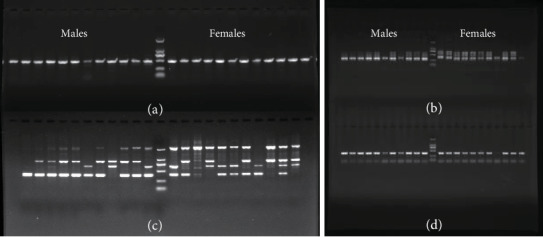
PCR amplifications of the four predicted male-specific ddRAD loci in *Eucommia ulmoides*. (a) MSL1. (b) MSL2. (c) MSL3. (d) MSL5. DNA ladder is in the middle of the panel with male-lanes on the left and female-lanes on the right.

**Figure 3:**
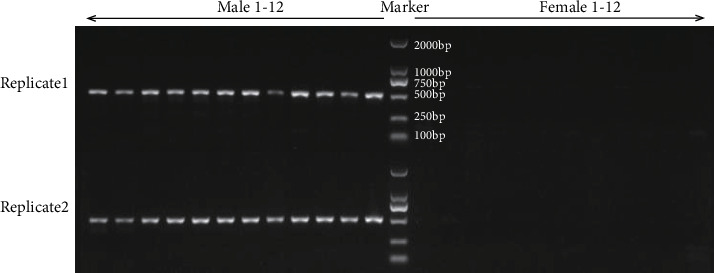
Agarose gel electrophoresis of PCR products of the male-specific ddRAD marker MSL4 in *Eucommia ulmoides*. Left lanes: male 1-12, right lanes: female 1-12. The result is robust in experimental replicates 1 and 2.

A total of 12 male individuals and 12 female individuals collected from the independent Henan population were used to examine the identified five candidate male-specific ddRAD loci (Table 2) by PCR amplification and Sanger sequencing. We found that none of the PCR gel banding of MSL1, MSL2, MSL3 and MSL5 were male-specific (Figure 2), even appearing no difference between the male and female ones (Figures 2(a), 2(b), and 2(d)). In contrast, the agarose gel electrophoresis of PCR products from the locus MSL4 displayed a distinct male-specific pattern, showing one single bright band with the expected size, about 500 bp, in each of the 12 male individuals whereas no banding in any of the 12 female individuals (Figure 3). Additionally, the results were consistent in two experimental replications.

**Figure 4:**
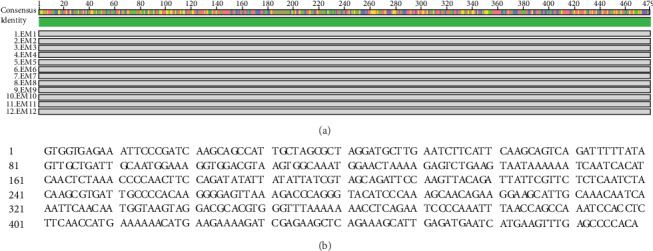
Sanger sequencing of the male-specific ddRAD marker MSL4 in *Eucommia ulmoides*. (a) Pairwise sequence alignments derived from MUSCLE program showing highly conserved MSL4 in males. The sequence identity is indicated on the top, and label EM1-12 represents the 12 males of *E. ulmoides* used for marker examination. **(**b) Consensus nucleotide sequence of the male-specific amplicon MSL4.

We further sequenced the male-specific band (MSL4) from 12 male individuals by Sanger sequencing. Sequences of MSL4 were highly conserved, with no sequence variations, in the 12 staminate genotypes (Figure 4(a)). The consensus sequence of this marker was determined as 479 bp in length (Figure 4(b)), as expected (circa 500 bp, Figure 3). Moreover, the produced 479 nucleotides matched perfectly with ddRAD sequences of MSL4 (a pair of 135 bp reads, Table 2), indicating the same genomic origin. The blast analysis found no hits for this male-specific ddRAD marker in the NCBI nucleotide database (https://www.ncbi.nlm.nih.gov/). Luckily, we detected a high similarity between the positions of 25,047-25,525 in GWHAAAL00024598#OriSeqID=scaffold571_obj#Len=331914 (*Eucommia* genome scaffolds, https://bigd.big.ac.cn/gwh/Assembly/13/show) and MSL4. The detailed results are as follows: Score = 835 bits (452), Expect = 0.0, Identities = 470/479 (98%), Gaps = 0/479 (0%), and Strand=Plus/Plus. We furthermore ascertained the annotation information of this scaffold (#OriSeqID=scaffold571_obj, Accession=GWHAAAL00024598) and found that MSL4 is probably non-protein-coding sequences located between CDS17 (24,544-24,670) and CDS18 (25,716-25,793) of a putative gene with unknown function.

## 4. Discussion

The evolution of dioecy has long been an intriguing issue since Darwin's time [50]. However, many dioecious plants have homomorphic sex chromosomes, e.g., wild strawberry (*Fragaria virginiana*) and garden asparagus (*A. officinalis*), which are difficult to be cytologically detectable for the lack of visible crossovers or of pairing [12, 51]. Until now, reports about the sex chromosomes of *E*. *ulmoides* are missing and gender discrimination by karyotype is undependable [21, 22]. Moreover, young and probably small sex-linked regions are intractable to be uncovered because they have stopped recombination during meiosis [19, 24]. Up to now, only a few of sex determination genes are dissected in *Actinidia*, *Asparagus*, *Diospyros*, and so on [10, 52]. Genetic sex determination with either male or female heterogamety in *E*. *ulmoides* is controversial [23]. Sex-specific molecular markers have been proven to be successful in identifying gender of seedlings, inferring sex determination system, and seeking sex determination genes [24, 51]. In this circumstance, we report a reproducible and consistent ddRAD-PCR method for sex identification of *E*. *ulmoides* based on the high-throughput sequencing technology.

Molecular markers such as SSR (simple sequence repeat), AFLP (amplified fragment length polymorphism), SCARs (sequence-characterized amplified regions) and RAPD (random amplification of polymorphic DNA) have been commonly used in exploring sex-specific markers of dioecious plants [34, 35]. However, a large amount of primers or primer pairs are required to screen sex-linked markers in these conventional methods, which are extremely labor intensive and time consuming. For example, a total of 1000 decamer primers were screened in order to identify a female-linked SCAR marker in *Humulus lupulus* [53]; in *A. officinalis*, 760 primers were used for developing a SCAR marker associated with the M (male-determining) locus [54]. Especially for tree species like *E*. *ulmoides*, it is even more difficult to screen sex-linked markers via conventional methods (e.g., SSR, AFLP, and RAPD) since experimental crosses and linkage map constructions are often impractical. For instance, in *P*. *vera* [55], only one 950 bp marker was found to be tightly linked to the female phenotype by screening 400 primers; a total of 45 decamer primers were examined for detecting a female-linked RAPD-SCAR detected in *H*. *rhamnoides* [56]. A previous study on *E*. *ulmoides* explored a pair of primers (EST-Eu059) out of 140 pairs of EST-SSR primers as sex-linked marker that displayed an extra band in male plants [57]. In contrast, the ddRAD-seq method used here generated a great number of genome-wide markers for *E*. *ulmoides* but with far less labor and cost. We obtained a high density of ddRAD tags for 20 males (183,752) and 20 females (147,122) of *E*. *ulmoides* (Table 1) across the genome, based on which five putative sex-specific markers were found (Table 2).

More importantly, ddRAD-seq technique in present study directly used multiple adult males and females in a natural population to screen sex-specific markers, which is largely different from previous studies exploring sex-linked markers based on experimental crosses and genetic segregation [39, 40, 58]. By bioinformatics analysis of ddRAD-seq data, we detected five candidate male-specific markers in *E*. *ulmoides* (Table 2). Based on further examination of these putative markers by PCR amplification in another independent population (Figures 2 and 3), we successfully identified a reliable sex-specific marker (MSL4) for PCR-based gender identification. The highly specialized banding pattern of MSL4 between males and females is very stable in different experimental replications (Figure 3). It is thus an ideal marker for sex identification of the seedling of *E*. *ulmoides* with simple PCR and agarose gel electrophoresis, similar to the SCAR markers developed for other dioecious species [35, 56]. Given that it is crucial to identify the gender of young trees for the agricultural industry in perennial woody plants with important economic values, just as the hardy rubber tree (*E. ulmoides*), this study provides a convenient and cost-effective method to screen sex-specific markers for gender discrimination. In addition to present study, the ddRAD-PCR technique has been proven solid in identifying sex-specific markers in the dioecious mangrove tree, *E. agallocha* [38] as well.

Gender identification of the young seedlings of *E*. *ulmoides* is substantially important in the breeding practice [59, 60]. As a tree species, *E*. *ulmoides* has to experience 8-10 years of juvenility period before flowering and fruiting [23]; therefore, early ascertaining the sex of seedlings can accelerate the artificial selection in breeding programs. The stable male-specific marker (MSL4) developed in this study is able to be used for rapid sex identification of *E*. *ulmoides* long before maturity when morphological characteristics of flowers are invisible. Additionally, this marker identified in the Shaanxi population of *E*. *ulmoides* also worked in the Henan population, which is suggesting that MSL4 probably is a universal marker for all populations of *E*. *ulmoides* across their national or global distributions. *E*. *ulmoides* has drawn much attention for hardy rubber (gutta) production during recent years [7, 61]. Previous studies have revealed sexual dimorphism in the leaf gutta content between males and females [21] and the highest gutta accumulation in fruit [23]. Therefore, it is essential to discern the sexes of seedlings of *E*. *ulmoides* with the molecular marker MSL4 to assure optimal sex ratio in *E*. *ulmoides* orchard, which will largely enhance their economic value.

Exploring sex-determining regions in nonmodel plant species may help us to understand the evolution of recombination suppression within young sex chromosomes [10, 62]. We sequenced the MSL4 fragment of 12 staminate genotypes from Henan population and found that there were no sequence variations at this locus (Figure 4(a)). Alignment of the 479 bp nucleotides (Figure 4(b)) and corresponding sequences from ddRAD-seq analysis (Table 2) further guaranteed a more convincing conclusion that they shared the same origin of the genome. In spite of null similarity hits of MSL4 in the NCBI nucleotide database (https://www.ncbi.nlm.nih.gov/), we luckily found a homologous sequence of MSL4 in the *Eucommia* genome database (GWHAAAL00024598, OriSeqID=scaffold571_obj, Len=331,914, https://bigd.big.ac.cn/gwh/Assembly/13/show) with nucleotide identities being 98% (positions: 25,047-25,525). It is noteworthy that the individual *E*. *ulmoides* plant used for genome sequencing was also a male tree [20], which enables us to identify the homologous sequences of MSL4, supporting the conclusion we draw here that the MSL4 is a male-specific marker. Three putative genes have been annotated with unknown functions in this scaffold (#OriSeqID=scaffold571_obj, Accession=GWHAAAL00024598, GWHAAAL00000000.gff), among which the MSL4 is situated in the intron between CDS17 (24,544-24,670) and CDS18 (25,716-25,793) of the first gene (2,411-25,793). Functional studies of this genomic locus will facilitate to unveil the sex determination mechanism of *E*. *ulmoides* in the future.

Sex-specific markers have also been used to infer sex determination mechanisms in several species, e.g., kiwifruit [13, 39] and papaya [63]. In this study, we identified five candidate male-specific markers and no female-specific loci using the ddRAD-seq technique (Table 2). Further PCR and Sanger sequencing examination demonstrated male-specific sequences at one of the five loci (Figures 3 and 4). The development of a male-specific fragment in a vast portion of the population suggests that the sex of *E*. *ulmoides* is probably determined genetically. Noteworthily, we only detected male-specific ddRAD markers but no female-specific loci, similar to a recent study that only found a male-specific SSR marker (EST-Eu059) in *E*. *ulmoides* [57]. The 479 bp male-specific locus may imply its location on sex chromosome, which suggests that *E*. *ulmoides* likely has an XX/XY sex determination system. But, we should keep it in mind that additional sex-linked markers need to be explored to dissect the genetic architecture of sex determination in *E*. *ulmoides* in the future.

## 5. Conclusions

In this study, we performed ddRAD-seq to screen putative sex-linked markers in the hardy rubber tree, *E. ulmoides*, based on which we then identified five candidate male-specific loci while no female-specific loci. One of the five male-specific loci (MSL4) was further validated by PCR amplification and Sanger sequencing in a geographically distant population. As a result, the male-specific ddRAD marker is a reproducible, quick, and precise tool for discriminating males from female seedlings in *E*. *ulmoides*. The results also indicated that *E*. *ulmoides* probably has a genetic sex determination system. Collectively, reliable gender identification of *E*. *ulmoides* at the juvenile stage with the male-specific marker is not only valuable for marker-assisted breeding program but also helpful to ascertain optimal sex ratio in *E*. *ulmoides* orchard to maximum increase gutta (Eu-rubber) yield. The combination method of ddRAD and PCR also shed light on identifying sex-specific markers in other dioecious tree species.

## Figures and Tables

**Table 1:** Samples of *Eucommia ulmoides* used for ddRAD-seq and summary of ddRAD-seq analysis for each sample.

Male	No. of reads	No. of ddRAD tags	Female	No. of reads	No. of ddRAD tags
M1	3,824,332	41,724	F1	3,621,226	47,001
M2	4,129,114	64,385	F2	2,253,982	23,616
M3	4,295,004	54,496	F3	5,256,178	79,809
M4	5,432,212	79,735	F4	4,352,006	64,904
M5	3,535,126	42,335	F5	6,369,882	92,210
M6	4,908,108	69,511	F6	4,485,857	56,084
M7	3,614,255	40,623	F7	2,816,328	30,228
M8	6,709,238	82,083	F8	4,107,792	56,497
M9	2,664,860	32,317	F9	3,809,033	46,428
M10	2,355,478	20,807	F10	2,727,482	24,611
M11	3,684,222	40,937	F11	5,165,036	82,508
M12	2,706,853	30,620	F12	5,593,874	76,306
M13	5,690,638	72,379	F13	6,251,392	95,993
M14	6,417,624	94,810	F14	3,956,216	38,078
M15	3,890,158	42,191	F15	6,330,162	90,762
M16	6,742,116	93,592	F16	4,312,874	63,217
M17	5,850,922	84,222	F17	5,295,320	78,552
M18	5,774,106	84,796	F18	3,544,489	35,002
M19	3,890,228	46,438	F19	4,686,812	65,568
M20	2,186,096	30,450	F20	5,504,118	84,643

**Table 2:** Putative male-specific ddRAD loci of *Eucommia ulmoides* predicted by bioinformatics analysis.

Locus	ddRAD sequences
MSL1	PE read 1
	GACCTAGGTACAGAAAATGTCCCCAATGAAATGAATGAAGTAGAAATATCAGCCGAAGCAGTGGAAGAGAAGATGTCAGATTGCCATGTGGATATGGAGACTGGTGTAGATGCTAACCCTAGTAACGGATCTGAA
	PE read 2
	GGTGTTCTAAATGAAGGTGTTGTTTTCTCCTCCGAAGTCTGTAGCAAGATAGAAGACTTTACAGTTAACGTGAAAACCCAACCTCAACAAGAGACAAGATAATCATCTTAGGTAATTTATACCCTCATGATAATA
MSL2	PE read 1
	GTCCTTAAAGTCCACGCATAGTCTTCACTTGTTTTTAGCCTTGGGTTCAATGACCAAGTTTGATATCCAATCCGTGAACTTCACCTTTTCTACTATATCGGACTTGATCAATTTGTTTATTTTTTCTTCAATGAT
	PE read 2
	GGGCACGGAGTACTAGAGCTCATGACCCTAGATTTTGAACCAGCATCGGTACCAATTAATAACCCTTGGGATGAAGAACCGACCCTCCTCCCTCATGAAGACCTTGTTAAAGTGTCCATCCACCCAACCATTAAG
MSL3	PE read 1
	GTCCTTCTCTGCATATTGACCGCCACGGCGGTGGTCTGGAAGCCTTAGCAGTAGGCTGCTTTTCAAAATTTGGCCCTTATGTTTGTTTTCTTATCGTTCTTCCACCATTCTTGATCTGATTTGATTGTTTATGAT
	PE read 2
	GGATTGGATTATTCACCAACAAAGCTCATAACTTAAACCATAAAGGTATAGGATATCCATATGTCAGATTGGGTTCCACTCTCCACTAATGTAGGCTTAGAAATTCAAAAAGATCAAAAGGACATGGATCGGTTT
MSL4	PE read 1
	GTCCCTTGATCTGCAAAAAGTAGACAGAGCCAACCAACAGGAAAAATTAAATAAATTAGTTGTGGTGAGAAATTCCCGATCAAGCAGCCATTGCTAGCGCTAGGATGCTTGAATCTTCATTCAAGCAGTCAGATT
	PE read 2
	GGTCGTTATTATTAAATATTTTATTTTTCTGTGTGATTAGCTTTTAGATGAATTGTGGGGCTCAAACTTCATGATTCATCTCAATGCTTTCTGAGCTTCTCGATCTTTTCTTCATGTTTTTTCATGGTTGAAGAG
MSL5	PE read 1
	GTCCCTTCCAAAGGAGATGAGAGAATAGGATATTTATAGGAAAAATATCTAAACAAAATATCTAATCTGTGCAGAGGTCACCGCCGCGGCGGTGAGTAGGTCGGTTGGTTGATCGCCTCAGCGGTGAAAAAGACG
	PE read 2
	GGTTTTGTTAAGTTTTAGAGCATTTTTATATGTTTTAATTAGTTTTTATTCTTTTTGTATTAAAATAGGTGTATGGGGCTAAAATATAGGAAACTTGAGCGAAAACATTGAGATCGAACCAAAACGGTCGAAGAA

## Data Availability

The ddRAD-seq data generated and analyzed in this study have been deposited in the Sequence Read Archive (SRA) of the NCBI database, with the accession number PRJNA607161.

## Supplementary Materials

Supplementary Materials Table S1: PCR primers used to amplify putative sex-linked ddRAD loci in Eucommia ulmoides.

## References

[1] Byng J. W., Chase M. W., Christenhusz M. J. (2016). An update of the Angiosperm Phylogeny Group classification for the orders and families of flowering plants: APG IV. *Botanical Journal of the Linnean Society*.

[2] Zhang Z. Y., Zhang H. D., Turland N. J., Wu Z. Y., Raven P. H., Hong D. Y. (2003). Eucommiaceae. *Flora of China*.

[3] Li F. D., Du H. Y. (2001). *Eucommia ulmoides*.

[4] Chen R., Harada Y., Bamba T., Nakazawa Y., Gyokusen K. (2012). Overexpression of an isopentenyl diphosphate isomerase gene to enhance trans-polyisoprene production in *Eucommia ulmoides* Oliver. *BMC Biotechnology*.

[5] Suzuki N., Uefuji H., Nishikawa T. (2012). Construction and analysis of EST libraries of the trans-polyisoprene producing plant, *Eucommia ulmoides* Oliver. *Planta*.

[6] Wang L., du H., Wuyun T. N. (2016). Genome-wide identification of microRNAs and their targets in the leaves and fruits of Eucommia ulmoides using high-throughput sequencing. *Frontiers in Plant Science*.

[7] Du H. Y., Hu W. Z., Yu R. (2015). *The Report on Development of China’s Eucommia Rubber Resources and Industry (1–2015)*.

[8] Nakazawa Y., Bamba T., Takeda T. (2009). Production of Eucommia-rubber from *Eucommia ulmoides* Oliv.(hardy rubber tree). *Plant Biotechnology*.

[9] Liu H. H., Du H. Y., Wuyun T. N. (2016). Advances in research on biotechnology breeding of Eucommia ulmoides. *Hunan Forestry Science and Technology*.

[10] Henry I. M., Akagi T., Tao R., Comai L. (2018). One hundred ways to invent the sexes: theoretical and observed paths to dioecy in plants. *Annual Review of Plant Biology*.

[11] Renner S. S. (2014). The relative and absolute frequencies of angiosperm sexual systems: dioecy, monoecy, gynodioecy, and an updated online database. *American Journal of Botany*.

[12] Ming R., Bendahmane A., Renner S. S. (2011). Sex chromosomes in land plants. *Annual Review of Plant Biology*.

[13] Fraser L. G., Tsang G. K., Datson P. M. (2009). A gene-rich linkage map in the dioecious species *Actinidia chinensis* (kiwifruit) reveals putative X/Y sex-determining chromosomes. *BMC Genomics*.

[14] Telgmann-Rauber A., Jamsari A., Kinney M. S., Pires J. C., Jung C. (2007). Genetic and physical maps around the sex-determining M-locus of the dioecious plant asparagus. *Molecular Genetics and Genomics*.

[15] Liu Z., Moore P. H., Ma H. (2004). A primitive Y chromosome in papaya marks incipient sex chromosome evolution. *Nature*.

[16] Nicolas M., Marais G., Hykelova V. (2004). A gradual process of recombination restriction in the evolutionary history of the sex chromosomes in dioecious plants. *PLoS Biology*.

[17] Yin T., DiFazio S. P., Gunter L. E. (2008). Genome structure and emerging evidence of an incipient sex chromosome in *Populus*. *Genome Research*.

[18] Błocka-Wandas M., Sliwinska E., Grabowska-Joachimiak A., Musial K., Joachimiak A. J. (2007). Male gametophyte development and two different DNA classes of pollen grains in *Rumex acetosa* L., a plant with an XX/XY1Y2 sex chromosome system and a female-biased sex ratio. *Sexual Plant Reproduction*.

[19] Charlesworth D. (2016). Plant sex chromosomes. *Annual Review of Plant Biology*.

[20] Wuyun T. N., Wang L., Liu H. (2018). The hardy rubber tree genome provides insights into the evolution of polyisoprene biosynthesis. *Molecular Plant*.

[21] Wang B., Wang Y., Mo H., Luo L., Li M., Cui K. (1999). Comparison of cytology, apical buds and gutta content between staminate and pistillate of Eucommia ulmoides trees. *Acta Botanica Sinica*.

[22] Zhang Y. D., He L. X., Li H., Cheng J. (2008). Karyotype analysis of *Eucommia ulmoides*. *Journal of Gasu Forestry Science and Technology*.

[23] Du H. Y. (2014). *China Eucommia Pictorial*.

[24] Zhang J., Boualem A., Bendahmane A., Ming R. (2014). Genomics of sex determination. *Current Opinion in Plant Biology*.

[25] Akagi T., Pilkington S. M., Varkonyi-Gasic E. (2019). Two Y-chromosome-encoded genes determine sex in kiwifruit. *Nature Plants*.

[26] Akagi T., Henry I. M., Tao R., Comai L. (2014). Plant genetics. A Y-chromosome-encoded small RNA acts as a sex determinant in persimmons. *Science*.

[27] Harkess A., Zhou J., Xu C. (2017). The asparagus genome sheds light on the origin and evolution of a young Y chromosome. *Nature Communications*.

[28] Wang J., Na J. K., Yu Q. (2012). Sequencing papaya X and Yh chromosomes reveals molecular basis of incipient sex chromosome evolution. *Proceedings of the National Academy of Sciences*.

[29] Geraldes A., Hefer C. A., Capron A. (2015). Recent Y chromosome divergence despite ancient origin of dioecy in poplars (*Populus*). *Molecular Ecology*.

[30] Blavet N., Blavet H., Muyle A. (2015). Identifying new sex-linked genes through BAC sequencing in the dioecious plant *Silene latifolia*. *BMC Genomics*.

[31] Wang W., Zhang X. (2017). Identification of the sex-biased gene expression and putative sex-associated genes in *Eucommia ulmoides* Oliver using comparative transcriptome analyses. *Molecules*.

[32] Zluvova J., Zak J., Janousek B., Vyskot B. (2010). Dioecious *Silene latifolia* plants show sexual dimorphism in the vegetative stage. *BMC Plant Biology*.

[33] Zemp N., Tavares R., Widmer A. (2015). Fungal infection induces sex-specific transcriptional changes and alters sexual dimorphism in the dioecious plant *Silene latifolia*. *PLoS Genetics*.

[34] Hale I., Melo A., Gustafson H. (2018). Sex-linked molecular markers for two cold-hardy kiwifruit species, *Actinidia arguta and A. kolomikta*. *European Journal of Horticultural Science*.

[35] Korekar G., Sharma R. K., Kumar R. (2012). Identification and validation of sex-linked SCAR markers in dioecious *Hippophae rhamnoides* L.(Elaeagnaceae). *Biotechnology Letters*.

[36] Andrews K. R., Good J. M., Miller M. R., Luikart G., Hohenlohe P. A. (2016). Harnessing the power of RADseq for ecological and evolutionary genomics. *Nature Reviews Genetics*.

[37] Yang G.-Q., Chen Y. M., Wang J. P. (2016). Development of a universal and simplified ddRAD library preparation approach for SNP discovery and genotyping in angiosperm plants. *Plant Methods*.

[38] Zhou Y., Wu W., Ning Z., Zhou R. (2018). Identification and characterization of sex-specific markers in the milky mangrove *Excoecaria agallocha* using double digest restriction site-associated DNA sequencing. *Aquatic Botany*.

[39] Zhang Q., Liu C., Liu Y. (2015). High-density interspecific genetic maps of kiwifruit and the identification of sex-specific markers. *DNA Research*.

[40] Qiu S., Bergero R., Guirao-Rico S. (2016). RAD mapping reveals an evolving, polymorphic and fuzzy boundary of a plant pseudoautosomal region. *Molecular Ecology*.

[41] Doyle J. J. (1987). A rapid DNA isolation procedure for small quantities of fresh leaf tissue. *Phytochemistry Bulletin*.

[42] Catchen J. M., Amores A., Hohenlohe P., Cresko W., Postlethwait J. H. (2011). Stacks: building and genotyping LociDe NovoFrom short-read *Sequences*. *Genetics*.

[43] Catchen J., Hohenlohe P. A., Bassham S., Amores A., Cresko W. A. (2013). Stacks: an analysis tool set for population genomics. *Molecular Ecology*.

[44] Andrews S. (2010). *FastQC: A Quality Control Tool for High Throughput Sequence Data*.

[45] Martin M. (2011). Cutadapt removes adapter sequences from high-throughput sequencing reads. *EMBnet Journal*.

[46] Fowler B. L., Buonaccorsi V. P. (2016). Genomic characterization of sex-identification markers in *Sebastes carnatus* and *Sebastes chrysomelas* rockfishes. *Molecular Ecology*.

[47] Koressaar T., Remm M. (2007). Enhancements and modifications of primer design program Primer3. *Bioinformatics*.

[48] Kearse M., Moir R., Wilson A. (2012). Geneious Basic: an integrated and extendable desktop software platform for the organization and analysis of sequence data. *Bioinformatics*.

[49] Altschul S. F., Gish W., Miller W., Myers E. W., Lipman D. J. (1990). Basic local alignment search tool. *Journal of Molecular Biology*.

[50] Barrett S. C. H. (2002). The evolution of plant sexual diversity. *Nature Reviews Genetics*.

[51] Charlesworth D. (2015). Plant contributions to our understanding of sex chromosome evolution. *New Phytologist*.

[52] Akagi T., Charlesworth D. (2019). Pleiotropic effects of sex-determining genes in the evolution of dioecy in two plant species. *Proceedings of the Royal Society B*.

[53] Polley A., Ganal M. W., Seigner E. (1997). Identification of sex in hop (*Humulus lupulus*) using molecular markers. *Genome*.

[54] Jiang C., Sink K. C. (1997). RAPD and SCAR markers linked to the sex expression locus M in asparagus. *Euphytica*.

[55] Hormaza J., Dollo L., Polito V. (1994). Identification of a RAPD marker linked to sex determination in *Pistacia vera* using bulked segregant analysis. *Theoretical and Applied Genetics*.

[56] Zhou W., Wang Y., Zhang G. (2018). Molecular sex identification in dioecious Hippophae rhamnoides L. via RAPD and SCAR markers. *Molecules*.

[57] Catchen J. M., Amores A., Hohenlohe P., Cresko W., Postlethwait J. H. (2016). development of gender-related EST-SSR markers in *Eucommia ulmoides*. *Scientia Silvae Sinicae*.

[58] Kafkas S., Khodaeiaminjan M., Güney M., Kafkas E. (2015). Identification of sex-linked SNP markers using RAD sequencing suggests ZW/ZZ sex determination in *Pistacia vera* L. *BMC Genomics*.

[59] Li Y., Wang D., Li Z., Wei J., Jin C., Liu M. (2014). A molecular genetic linkage map of *Eucommia ulmoides* and quantitative trait loci (QTL) analysis for growth traits. *International Journal of Molecular Sciences*.

[60] Wang D., Li Y., Li L., Wei Y., Li Z. (2014). The first genetic linkage map of *Eucommia ulmoides*. *Journal of Genetics*.

[61] Wang W., Chen S., Zhang X. (2018). Whole-genome comparison reveals heterogeneous divergence and mutation hotspots in chloroplast genome of *Eucommia ulmoides* Oliver. *International Journal of Molecular Sciences*.

[62] Renner S. S. (2016). Pathways for making unisexual flowers and unisexual plants: moving beyond the “two mutations linked on one chromosome” model. *American Journal of Botany*.

[63] Ming R., Yu Q., Moore P. H. (2007). Sex determination in papaya. *Seminars in cell & developmental biology*.

