# Supplementary material for: Molecular Sex Identification in the Hardy Rubber Tree (Eucommia ulmoides Oliver) via ddRAD Markers
Source: Int J Genomics. 2020 May 16;2020:2420976. doi: 10.1155/2020/2420976 (PMC7246395; doi:10.1155/2020/2420976)
Supplement: Supplementary Materials — Table S1: PCR primers used to amplify putative sex-linked ddRAD loci in Eucommia ulmoides. [file 2420976.f1.doc]

**Table S1.** PCR primers used to amplify putative sex-linked ddRAD loci in *Eucommia ulmoides.*

| **Locus** | **Primer sequences (5'-3')** |
| --- | --- |
| MSL1 |  |
| MSL1-F | CCGAAGCAGTGGAAGAGAAG |
| MSL1-R | CTCCTCCGAAGTCTGTAGCA |
| MSL2 |  |
| MSL2-F | AGTCCACGCATAGTCTTCAC |
| MSL2-R | CCTCCTCCCTCATGAAGACC |
| MSL3 |  |
| MSL3-F | CTCTGCATATTGACCGCCAC |
| MSL3-R  MSL4  MSL4-F  MSL4-R  MSL5  MSL5-F  MSL5-R | TGGGTTCCACTCTCCACTAA  ACAGAGCCAACCAACAGGAA  TGTGGGGCTCAAACTTCATG  GTCCCTTCCAAAGGAGATGA  GAACCAAAACGGTCGAAGAA |
